# Improving effect of microendoscopic decompression surgery on low back pain in patients with lumbar spinal stenosis and predictive factors of postoperative residual low back pain: a single-center retrospective study

**DOI:** 10.1186/s12891-021-04844-y

**Published:** 2021-11-15

**Authors:** Ryo Taiji, Hiroshi Iwasaki, Hiroshi Hashizume, Yasutsugu Yukawa, Akihito Minamide, Yukihiro Nakagawa, Shunji Tsutsui, Masanari Takami, Keiji Nagata, Shizumasa Murata, Takuhei Kozaki, Munehito Yoshida, Hiroshi Yamada

**Affiliations:** 1 Department of Orthopaedic Surgery, Wakayama Medical University, 811-1 Kimiidera, Wakayama, 641-8510 Japan; 2 Spine Center, Department of Orthopaedic Surgery, Dokkyo Medical University Nikko Medical Center, 632 Takatoku, Tochigi, 321-2593 Japan; 3 Spine Care Center, Wakayama Medical University Kihoku Hospital, 219 Myoji, Katsuragi-cho, Wakayama, 649-7113 Japan; 4 Sumiya Orthopaedic Hospital, 337 Yoshida, Wakayama, 640-8343 Japan

**Keywords:** Lumbar spinal stenosis, Decompression surgery, Spinal endoscopy, Low back pain, Surgical treatment

## Abstract

**Background:**

Although there are reports on the effectiveness of microendoscopic laminotomy using a spinal endoscope as decompression surgery for lumbar spinal stenosis, predicting the improvement of low back pain (LBP) still poses a challenge, and no clear index has been established. This study aimed to investigate whether microendoscopic laminotomy for lumbar spinal stenosis improves low back pain and determine the preoperative predictors of residual LBP.

**Methods:**

In this single-center retrospective study, we examined 202 consecutive patients who underwent microendoscopic laminotomy for lumbar spinal stenosis with a preoperative visual analog scale (VAS) score for LBP of ≥40 mm. The lumbar spine Japanese Orthopaedic Association (JOA), and VAS scores for LBP, leg pain (LP), and leg numbness (LN) were examined before and at 1 year after surgery. Patients with a 1-year postoperative LBP-VAS of ≥25 mm composed the residual LBP group. The preoperative predictive factors associated with postoperative residual LBP were analyzed.

**Results:**

JOA scores improved from 14.1 preoperatively to 20.2 postoperatively (*p* < 0.001), LBP-VAS improved from 66.7 to 29.7 mm (*p* < 0.001), LP-VAS improved from 63.8 to 31.2 mm (*p* < 0.001), and LN-VAS improved from 63.3 to 34.2 mm (*p* < 0.001). Ninety-eight patients (48.5%) had a postoperative LBP-VAS of ≥25 mm. Multiple logistic regression analysis revealed that Modic type 1 change (odds ratio [OR], 5.61; 95% confidence interval [CI], 1.68–18.68; *p* = 0.005), preoperative VAS for LBP ≥ 70 mm (OR, 2.19; 95% CI, 1.17–4.08; *p* = 0.014), and female sex (OR, 1.98; 95% CI, 1.09–3.89; *p* = 0.047) were preoperative predictors of residual LBP.

**Conclusion:**

Microendoscopic decompression surgery had an ameliorating effect on LBP in lumbar spinal stenosis. Modic type 1 change, preoperative VAS for LBP, and female sex were predictors of postoperative residual LBP, which may be a useful index for surgical procedure selection.

## Background

Lumbar spinal stenosis (LSS) is a disease in which the dural tract and nerve roots are compressed because of degeneration of the intervertebral disk and facet joints, causing symptoms such as sciatica, intermittent claudication, and low back pain (LBP) [1, 2]. The number of patients suffering from LSS is expected to increase further in the future due to an aging population. Most patients with LSS initially receive conservative treatment to manage their symptoms. Surgery is an effective treatment option when conservative methodologies fail [2, 3]. Generally, decompression surgery is performed when there is associated lower limb pain and gait disturbance, and fusion surgery is adopted for patients with LBP, spondylolisthesis, lumbar instability, and scoliosis [4, 5].

Microendoscopic laminotomy (MEL) is a minimally invasive surgical procedure that incorporates the use of a spinal endoscope. This procedure, which is derived from the microendoscopic discectomy technique, was developed to treat patients with LSS [6, 7]. The advantages of this procedure include a small incision, excellent visualization, gentle tissue dissection, and bilateral decompression via a unilateral tubular approach. MEL has been reported to be effective for patients with LSS with/without degenerative spondylolisthesis. Additionally, MEL has been shown to provide a good Japanese Orthopedic Association (JOA) score recovery rate, improvement of LSS symptoms, as indicated by the Roland-Morris Disability Questionnaire, and short-form 36 scores at 5-year follow-ups [7].

In the procedure performed to improve lower limb symptoms, patients often report an improvement in LBP. It has been reported that open decompression surgery has an ameliorating effect on LBP [8]. However, it is difficult to predict the improvement of LBP with decompression surgery. The purpose of this study was to investigate whether MEL, a motion-preserving decompression surgery that uses a spinal endoscope, improves LBP with LSS, and to determine the preoperative predictors of residual LBP after surgery.

## Methods

This retrospective cohort study was approved by institutional review board of Wakayama Medical University (No. 2945). Written informed consent for involvement in this study was obtained from all patients included. In total, 220 consecutive patients who underwent MEL for LSS between July 2014 and December 2018 were enrolled. Patients who underwent microendoscopic discectomy for lumbar disc herniation were excluded from this study. The LSS diagnoses were made based on the presence of specific clinical symptoms, such as LBP, leg pain, numbness, and intermittent claudication, as well as on the existence of radiological spinal stenosis on magnetic resonance imaging (MRI) that confirmed the neurological symptoms. The inclusion criteria were as follows: (1) patients who underwent MEL for LSS due to failure of conservative treatment, (2) patients who had a preoperative baseline LBP of at least 40 mm on the 100-mm visual analog scale (VAS). Patients were excluded if they had a history of lumbar fusion surgery.

**Fig. 1:**
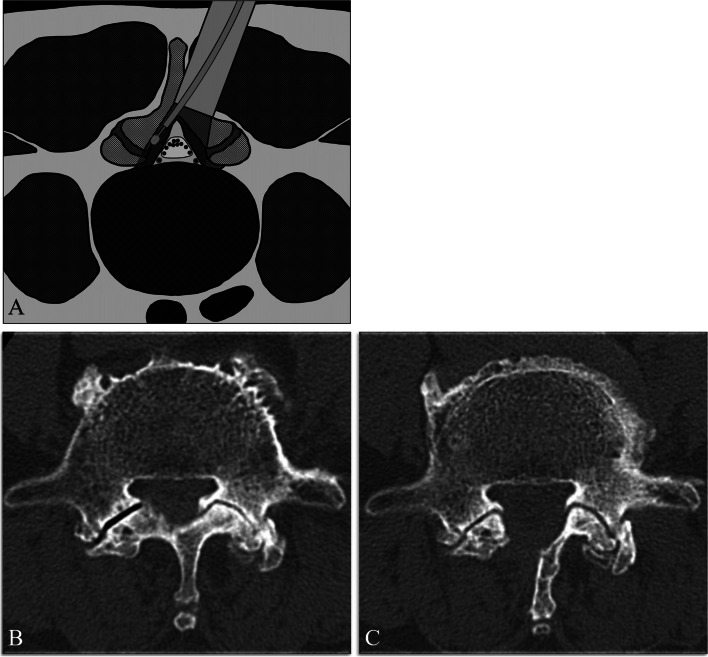
**A** Microendoscopic laminotomy (MEL) schema. **B** and **C** Representative axial computed tomography (CT) images obtained preoperatively (**B**) and after decompression by MEL (**C**)

The surgeries were performed using a METRx endoscopic system (Medtronic Sofamor Danek, Memphis, TN) by 11 spine surgeons who were certified as endoscopic spine surgeons by the Japanese Orthopedic Association. To perform the spinal decompression, a small paramedian skin incision of approximately 16 mm in length was made to target the interlaminar space in each decompressed level. The central canal and bilateral lateral recess were decompressed for all the patients through a unilateral tubular approach (Fig. 1). While performing the decompression, the facet joints were preserved using a high-speed drill with a long curved endoscopic bit and curved Kerrison rongeurs, as previously reported [7].

As clinical outcomes, the lumbar spine Japanese Orthopaedic Association (JOA) score and VAS scores for LBP, leg pain, and leg numbness were examined before and at 1 year after surgery. The minimal clinically important difference (MCID) of the VAS value improvement was determined as ≥20 mm [9], and the achievement rate of the MCID for LBP improvement was investigated.

Subsequently, for the purpose of examining preoperative predictors of postoperative residual LBP, patients with a 1-year postoperative LBP-VAS of ≥25 mm or those requiring additional surgery for residual LBP within 1 year after MEL surgery were considered to have postoperative residual LBP. The factors that may be associated with postoperative residual LBP, including age, sex, body mass index (BMI), disease duration, number of decompressed levels, and preoperative clinical scores (VAS scores) were investigated. Preoperative radiological findings, such as spondylolisthesis (Meyerding classification), degenerative scoliosis (Cobb angle ≥20°), spinal instability (anterior translation of the affected motion segment exceeding 10% of the AP vertebral depth or intervertebral rotation exceeding 10° [10]), intervertebral disk degeneration (Pfirrmann classification), endplate disorders (Modic changes [11]), disk space vacuum phenomenon, old vertebral fractures, and sagittal malalignment (sagittal modifiers [12]) were also evaluated. Spondylolisthesis, scoliosis, spinal instability, old vertebral fractures, and sagittal malalignment were measured using plain radiographs, while intervertebral disc degeneration and endplate disorders were evaluated on MRI and disk space vacuum phenomenon was evaluated by computed tomography. Spinal instability, intervertebral disk degeneration, endplate disorders, and disk space vacuum phenomenon were all assessed at the most severe of the surgically treated intervertebral levels. All the measurements were performed by another spine surgeon who was not involved in the surgeries.

### Statistical analysis

We performed a univariate analysis to assess whether these factors were related to postoperative residual LBP. The Student’s t-test was used to examine differences in age, BMI, disease period, and VAS scores for LBP, leg pain, and leg numbness between patients with postoperative VAS for LBP ≥ 25 mm as compared to postoperative VAS for LBP < 25 mm. The Chi-square test was used to examine the differences in sex, number of decompressed levels, spondylolisthesis, degenerative scoliosis, spinal instability, disk degeneration, endplate disorder, vacuum phenomenon of disk space, old vertebral fractures, sagittal malalignment, and preoperative VAS scores for LBP between patients with postoperative VAS for LBP ≥ 25 mm and those with postoperative VAS for LBP < 25 mm. The preoperative VAS for LBP was evaluated as 2 values by setting a threshold value through a simple logistic analysis. We performed multiple logistic regression analyses using the residual LBP as the dependent variable, with explanatory variables including factors that showed a *p* value of < 0.2 in the univariate analysis and factors that have been reported as poor factors for decompression surgery (spondylolisthesis [13, 14], intervertebral instability [15], scoliosis [16], preoperative LBP [4, 5]). The Hosmer-Lemeshow test was performed for the suitability of multiple logistic models.

Statistical analyses were performed using JMP Pro 13 (SAS Institute Japan, Tokyo, Japan) and SPSS Statistics Ver. 28 (SPSS Inc., Chicago, IL). *P* values < 0.05 were considered statistically significant.

## Results

Altogether, 202 patients (males, *n* = 109; females, *n* = 93: follow-up rate, 91.8%) who had been observed for > 1 year after surgery were enrolled. Table 1 shows the demographic data of all patients. Patients’ average age at surgery was 72.4 years (range, 51–91 years). The average follow-up period was 29.8 months (range, 12–64 months). All the patients had neurogenic claudication. A total of 134 patients had radicular pain, 119 had bilateral leg numbness, and 51 experienced both symptoms. A total of 170 patients underwent surgery for central stenosis, and the other 32 patients were treated for lateral recess stenosis. In addition, 98 of the patients had spondylolisthesis. There were 105 patients with single-level, 71 with two-level, 23 with three-level, and three with four-level decompression.

**Table 1:** Summary of the patients’ demographic and clinical characteristics

	n (%)
**Overall**	202 (100)
**Age** (years)	
≤ 59	24 (11.9)
60–69	49 (24.3)
70–79	76 (37.6)
≥ 80	53 (26.2)
**Sex**	
Male	109 (54.0)
Female	93 (46.0)
**Number of levels**	
1	105 (52.0)
2	71 (35.1)
3	23 (11.4)
4	3 (1.5)
**Operated level**	
L1/2	3 (1.5)
L2/3	39 (19.3)
L3/4	107 (53.0)
L4/5	160 (79.2)
L5/S	20 (9.9)

### Improvement of LBP

The JOA score improved from 14.1 preoperatively to 20.2 postoperatively (*p* < 0.001), VAS score for LBP improved from 66.7 mm to 29.7 mm (*p* < 0.001), VAS score for leg pain improved from 63.8 mm to 31.2 mm (*p* < 0.001), and VAS score for leg numbness improved from 63.3 mm to 34.2 mm (*p* < 0.001) (Table 2). Apart from the JOA score and VAS score for leg pain, VAS score for LBP also significantly improved. The MCID for the improvement of VAS score for LBP was achieved in 148 patients (73.3%).

**Table 2:** Surgical outcomes assessed by clinical scores

	Before surgery	1 Year after surgery	*P*-value
JOA score	14.1 ± 4.3	20.2 ± 5.0	< 0.001
VAS			
Low back pain	66.7 ± 15.9	29.7 ± 28.8	< 0.001
Leg pain	63.8 ± 22.0	31.2 ± 31.4	< 0.001
Leg numbness	63.3 ± 26.6	34.2 ± 33.4	< 0.001

Values are presented as means and standard deviations

*JOA* Japanese Orthopaedic Association, *VAS* Visual analog scale

### Predictive factors for residual LBP

At 1 year after surgery, 98 patients (including five who underwent salvage with fusion surgery) had a VAS for LBP ≥ 25 mm. Factors that showed a *p* value of < 0.2 in the univariate analysis were female sex (*p* = 0.034), BMI (*p* = 0.141), scoliosis (*p* = 0.003), Modic type 1 change (*p* ≤ 0.001), pelvic tilt ≥30° (*p* = 0.113), and preoperative VAS for LBP ≥ 70 mm (*p* = 0.001) (Table 3).

**Table 3:** Demographic characteristics and preoperative radiological findings and clinical scores

	Postoperative VAS for LBP ≥ 25 mm (*n* = 98)	Postoperative VAS for LBP < 25 mm (*n* = 104)	*P*-value
Age (years)	72.3 ± 9.1	72.6 ± 9.0	0.845
Sex			0.034*
Male	45.9%	61.5%	
Female	54.1%	38.5%	
BMI (kg/m^2^)	24.9 ± 4.0	24.1 ± 3.7	0.141
Disease period (months)	33.4 ± 33.2	31.4 ± 32.6	0.681
Number of levels			0.645
1	55.1%	49.0%	
2	33.7%	36.5%	
≥ 3	11.2%	14.4%	
**Preoperative radiological findings**			
Spondylolisthesis			
≥ Grade 1	46.9%	50.0%	0.675
≥ Grade 2	8.2%	3.9%	0.241
Scoliosis	13.3%	1.9%	0.003*
Spinal instability	18.4%	17.5%	1.000
Disk degeneration			
Grades 1, 2, 3	7.1%	8.7%	0.797
Grade 4	59.2%	62.5%	0.667
Grade 5	33.7%	28.9%	0.544
Modic endplate changes			
Type 1	21.4%	3.9%	< 0.001*
Type 2	7.1%	14.4%	0.116
Type 3	2.0%	0%	0.234
Vacuum phenomenon of disc space	68.4%	64.4%	0.653
Old vertebral fractures	12.2%	10.6%	0.826
Sagittal parameters			
PI-LL ≥ 10°	62.9%	60.6%	0.773
PI-LL ≥ 20°	37.1	31.7%	0.459
SVA ≥ 40 mm	61.2%	63.5%	0.773
SVA ≥ 95 mm	21.4%	18.3%	0.600
PT ≥ 20°	60.8%	51.0%	0.201
PT ≥ 30°	24.7%	15.4%	0.113
**Preoperative clinical scores**			
VAS for low back pain	70.1 ± 15.9	63.4 ± 15.3	0.003*
VAS for LBP ≥ 70 mm	55.1%	31.7%	0.001*
VAS for leg pain	64.2 ± 24.0	63.4 ± 20.1	0.795
VAS for leg numbness	65.2 ± 27.9	61.4 ± 25.2	0.315

Values are presented as means and standard deviations or percentages

*p < 0.05

*LBP* Low back pain, *BMI* Body mass index, *PI-LL* Pelvic incidence minus lumbar lordosis, *SVA* Sagittal vertical axis, *PT* Pelvic tilt, *VAS* Visual analog scale

Multiple logistic regression analyses showed that Modic type 1 change (odds ratio [OR], 5.61; 95% confidence interval [CI], 1.68–18.68; *p* = 0.005), preoperative VAS for LBP ≥ 70 mm (OR, 2.19; 95% CI, 1.17–4.08; *p* = 0.014), and female sex (OR, 1.98; 95% CI, 1.09–3.89; *p* = 0.047) were predictors of postoperative residual LBP (Table 4). On the other hand, spondylolisthesis, intervertebral instability, and sagittal malalignment were not predictors of LBP residuals.

**Table 4:** Predictors of postoperative residual low back pain

	Odds ratio	95% CI	*P*-value
Sex (male, 1 vs female, 0)	1.98	1.09–3.89	0.047*
Modic type 1 (presence, 1 vs absence, 0)	5.61	1.68–18.68	0.005*
Preoperative VAS for LBP ≥ 70 mm (yes, 1 vs no, 0)	2.19	1.17–4.08	0.014*

*VAS* Visual analog scale, *LBP* Low back pain

*p < 0.05

## Discussion

In the selection of a surgical procedure for LSS, it has been reported that decompression alone yields inferior results compared with fusion surgery in patients with LBP [4, 5] or radiological abnormalities, such as spondylolisthesis and intervertebral instability [14–16]. For this reason, fusion surgery has been widely accepted as a surgical treatment for LSS with LBP. However, some studies have reported that decompression surgery via the open method performed for improving lower limb pain also has a secondary effect on ameliorating LBP [8, 17]. This suggests that decompression alone may provide satisfactory results in some LSS patients with LBP. In this study, the average VAS score of patients whose VAS score for LBP was ≥40 mm before surgery improved from 66.7 mm before surgery to 29.7 mm after surgery. The MCID for improvement of VAS score was achieved in > 70% of the patients. This result highlights the effect of MEL on LBP improvement. The mechanism by which decompression surgery reduces LBP with LSS includes (1) the direct effects on radicular LBP by decompression of the cauda equina and nerve roots, (2) improvement of LBP associated with leg pain-alleviating posture improvements, (3) denervation around facet joints by a surgical procedure, (4) improvement of physical function or gait disturbance, and (5) psychological effects [8, 18]. However, it is difficult to isolate the exact cause in cases where many factors are involved. The surgical technique used in the present study employed a spinal endoscope for decompression without damaging the facet joints and paravertebral muscles. Therefore, it is difficult to consider the effect of denervation around the facet joint as an improvement mechanism for LBP in the present study. Consequently, it is presumed that these causes are due to nerve decompression or postural changes via MEL; however, further investigation is needed to clarify this mechanism.

As per the preoperative predictive factors, patients were divided according to a postoperative LBP VAS score of 25 mm. We adopted the VAS score of 25 mm, since the pain level less than that value is reported as acceptable pain for degenerative disorders [19]. In the univariate analysis, female sex, BMI, scoliosis, Modic type 1 change, pelvic tilt (≥30°), and preoperative VAS for LBP were identified as predictors of postoperative residual LBP. In the multiple logistic regression analyses, Modic type 1 change of endplate, preoperative VAS for LBP, and female sex remained predictors of postoperative residual LBP. No problem with multicollinearity was noted because all variance inflation factors are < 10. The Hosmer-Lemeshow test was performed for the suitability of the multiple logistic model, and no problems were observed.

Modic type 1 endplate disorder is a change in T1 low intensity and T2 high intensity, which reflects inflammatory findings [11], and its association with LBP has been reported [20]. LBP has been related to histological abnormalities, such as inflammatory cytokines and nerve ingrowth into the vertebral endplates [21, 22], and it is reasonable that decompression alone does not improve the pain. Preoperative VAS for LBP was also associated with residual LBP. In the present study, a model was created by converting the preoperative VAS for LBP into two values with a threshold of 70 mm derived by a simple logistic analysis to make it clinically useful. However, since the area under the receiver operating characteristic curve is 0.62, it should be determined whether or not this threshold is appropriate. Either way, indications for decompression surgery may need to be carefully considered for patients with severe preoperative low back pain. Female sex was also associated with residual LBP. In the postoperative clinical results of LSS, it has been reported that female sex has a negative effect on LBP, lower limb pain, and the Oswestry disability index [4, 23]. The results of this study were consistent with those of previous reports. However, the coexistence of osteoporosis or a decrease of the trunk muscle mass, which was not investigated in the present study, may affect the association between female sex and residual LBP [24, 25]. Moreover, it should be noted that women in the general population are more likely to have LBP [26, 27]. Therefore, we believe that the results of this study do not necessarily indicate the need to limit the indications of decompression surgery for women. On the other hand, the morphological abnormalities such as spondylolisthesis, spinal instability, and sagittal malalignment were not related to postoperative residual LBP in the present study. One factor that contributed to the conflicting results between the present study and previous reports may be that MEL was superior to conventional open decompression in preserving posterior tissues, such as paravertebral muscles and facet joints. This result suggests that there is no need to select fusion surgery just due to these abnormalities in imaging findings, and there are patients in whom satisfactory results can be obtained only by decompression surgery.

There are some limitations in this study. First, this is a retrospective cohort study with a small number of patients. Future prospective studies with a larger patient population are needed. Second, the follow-up period in this study was short. However, since it is intended for LBP, which can be affected by multiple factors, we believe that the evaluation of short-term postoperative results is appropriate for the purpose of clearly evaluating the improving effect of surgery. Third, there is a risk of selection bias, and there is no comparison with fusion surgery. However, this study includes a considerable number of patients with spondylolisthesis or spinal instability, and there have been multiple cases of LBP improvement with decompression surgery alone. These results may provide valuable information for patients undergoing surgery.

## Conclusions

Microendoscopic decompression surgery for LSS has an ameliorating effect on LBP. Modic type 1 change of endplate, preoperative VAS for LBP, and female sex are predictors of postoperative residual LBP, which may be a useful index for surgical procedure selection. There is no need to select fusion surgery for these cases due to imaging abnormalities, such as spondylolisthesis, spinal instability, and sagittal malalignment.

## Data Availability

The datasets used and/or analysed during the current study are available from the corresponding author on reasonable request.

## Abbreviations

MEL: Microendoscopic laminotomy
LSS: Lumbar spinal stenosis
LBP: Low back pain
VAS: Visual analogue scale
JOA: Japanese Orthopaedic Association
BMI: Body mass index
